# A possible role of plasmin-dependent activation of TGF-β in cancer-associated thrombosis: Implications for therapy

**DOI:** 10.1007/s10555-024-10222-6

**Published:** 2024-11-22

**Authors:** Marta Smeda, Ebrahim H. Maleki, Agnieszka Jasztal

**Affiliations:** 1 Jagiellonian Centre for Experimental Therapeutics (JCET), Jagiellonian University, Bobrzynskiego 14, Krakow, 30-348 Poland; 2 Doctoral School of Exact and Natural Sciences, Jagiellonian University, Krakow, Poland

**Keywords:** Cancer-associated thrombosis, Anticoagulant/antiplatelet treatment, TGF-β, Plasmin activity, PAI1

## Abstract

While the prevalence of cancer-associated thrombosis (CAT) is high in cancer patients, its molecular mechanisms have not been fully elucidated. Moreover, the risks of recurrent CAT events and mortality remain high in cancer patients despite the introduction of anticoagulant/antiplatelet therapy. Here, we discuss the possibility that increased plasmin activity driven by anticoagulant/antiplatelet treatment might be the major mechanism responsible for the activation of an excess of cancer-derived transforming growth factor-beta (TGF-β) originating from cancer cells and the tumour microenvironment. Hence, high coagulation and fibrinolysis rates in cancer patients may be linked to high rates of TGF-β activation, especially the excess of TGF-β derived from cancer cells. In turn, high TGF-β activation could contribute directly to maintaining high thrombotic risk and CAT recurrence in cancer patients since TGF-β signalling increases gene expression and secretion of the fibrinolysis inhibitor plasminogen activator inhibitor 1 (PAI1). Thus, TGF-β could directly contribute to the high number of deaths among patients with cancer experiencing CAT, despite anticoagulant/antiplatelet treatment. In a longer-term perspective, increased TGF-β activation, by supporting a pro-coagulant cancer microenvironment, might also accelerate cancer progression. This review aims to discuss the published evidence that might support the scenario described above, and to put forward the hypothesis that cancer patients experiencing CAT events would largely benefit from anti-TGF-β therapy.

## Cancer-associated thrombosis

Among patients with cancer, although the most frequent non-cancer cause of death is infection [1], cancer-associated thrombosis (CAT) is another leading cause of mortality [2]. It is estimated that among the 20 million new cancer cases in 2022, even up to 20% of them (about 4 000 000 people) could experience thrombotic events at some stage [3, 4], the rate being highest in the initial period following diagnosis [5]. These numbers will rise in the future, with over 35 million annual new cancer cases predicted by 2050 [3].

Until now, many mechanistic explanations of CAT have been proposed, such as the overactivation of coagulation pathways by circulating cancer cells [6], anticancer therapy [7], the immobilisation of patients with cancer [8], the patient’s age (patients aged 60 years or older have a higher incidence of CAT) [8], and prior history of thrombotic events (patients with a history of venous thromboembolism (VTE) have up to 6-fold increased risk of VTE recurrence) [7, 8]. Finally, the risk of CAT strongly depends on cancer-associated risk factors such as the site of the primary tumour, with pancreatic cancer being the most frequently linked to CAT incidence [5], and the stage of the disease, with patients harbouring advanced cancers with distant metastases being at higher risk of CAT events compared to patients without distant metastases [7, 8].

Among the most widely-recognised thrombotic complications is VTE, which manifests as deep vein thrombosis or pulmonary embolism [2]. The risk of VTE was recently estimated to be 85% higher in patients with cancer and 91% higher in those receiving chemotherapy or targeted therapy compared to the general population [7]. In particular, the risk of VTE is increased in cancer patients receiving immunotherapy [9]. Arterial thromboembolism (ATE) can also be related to cancer progression (but is less common than VTE) and includes the common forms of myocardial infarction or stroke [10]. Like VTE, ATE occurs more frequently in patients with cancer than without [10]. Finally, CAT can manifest with conditions such as disseminated intravascular coagulation (DIC) [11] or thrombotic microangiopathy (TMA) [12].

Despite the high prevalence of CAT events in cancer patients, its molecular mechanisms are poorly understood. Currently, CAT is thought to be driven directly by cancer cells (expressing or releasing procoagulant agents) or by indirect mechanisms depending on the cancer-driven procoagulant phenotype of other cells [13]. In the former, cancer cells release tissue factor (TF) and the fibrinolysis inhibitor plasminogen activator inhibitor 1 (PAI1), express podoplanin (PDPN), which binds to and activates the C-type lectin-like receptor 2 (CLEC2) on platelets, and expose phosphatidylserine that promotes coagulation by forming a surface for coagulation complexes. In contrast, the indirect mechanisms depend on inflammatory or angiogenic cytokines such as vascular endothelial growth factor (VEGF) and tumour necrosis factor-alpha (TNF-α).

## The TGF-β cytokine in regulation of fibrinolysis and cancer progression

**Fig. 1:**
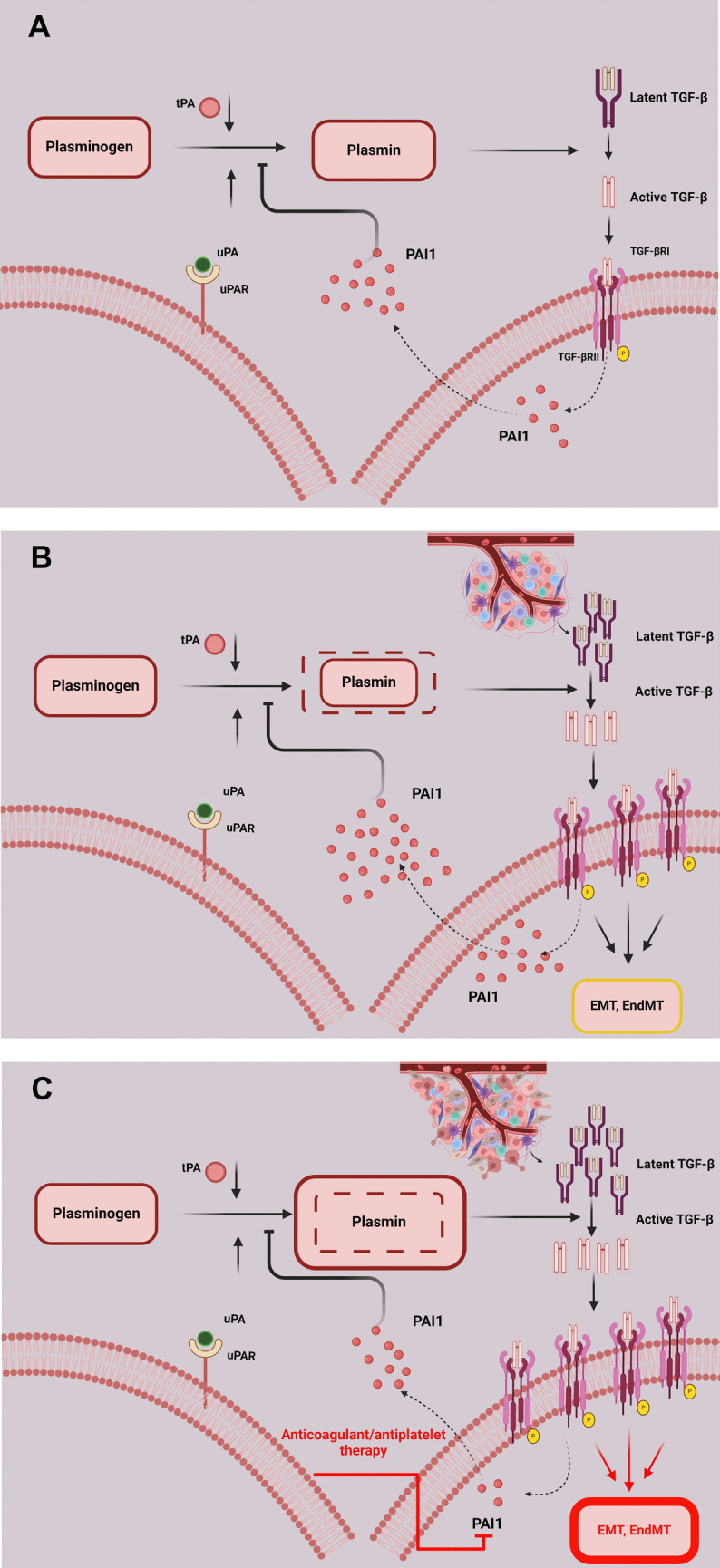
Plasmin-dependent activation of latent TGF-β in health and cancer progression. ( **A **) tPA (tissue plasminogen activator) and uPA (urokinase plasminogen activator) convert plasminogen to plasmin, thereby activating the process of fibrinolysis after thrombus formation. However, plasmin also releases active TGF-β from its latent complex. Active TGF-β triggers, via its receptors TGF-βRII and RI, synthesis and secretion of plasminogen activator inhibitor 1 (PAI1) that constrains plasmin generation. In this way, PAI1 prevents excessive TGF-β activation in a self-regulatory loop previously described by Khalil N [ 14 ]. ( **B **) Increased generation of the active form of TGF-β in cancer potentiates TGF-β-dependent PAI1 synthesis and secretion to counteract excessive TGF-β activation and signalling. Nevertheless, increased levels of PAI1 also inhibit fibrinolysis, increasing thrombotic risk. ( **C** ) Introduction of anticoagulant/antiplatelet therapy lowers PAI1 levels and increases fibrinolysis rates allowing for higher plasmin generation. This also increases plasmin-dependent activation of TGF-β secreted from cancer cells and the tumour microenvironment

TGF-β signalling is involved in the control of fibrinolysis rates since TGF-β drives expression and secretion of fibrinolysis inhibitor PAI1 [14] (Fig. 1A). After thrombus formation, tissue-type plasminogen activator (tPA) and urokinase plasminogen activator (uPA) convert plasminogen into plasmin to activate fibrinolysis. However, simultaneously, plasmin releases active TGF-β from its latent complex and TGF-β signalling triggers PAI1 synthesis and secretion that inhibits further plasminogen conversion into plasmin to counteract excessive activation of TGF-β. Therefore, rates of fibrinolysis and plasmin-dependent activation of TGF-β are closely linked, both positively regulated by plasmin and inhibited by PAI1. In physiological conditions, activation of latent TGF-β by plasmin is self-limiting until homeostatic balance is achieved in plasmin activity, the amount of TGF-β, and the levels of PAI-1 [14].

In conditions of cancer (Fig. 1B), different types of tumour cells and the tumour microenvironment generate large amounts of TGF-β which differ not only among tumour types but can also vary significantly among patients suffering from the same cancer type. For instance, in a population of 51 patients undergoing resection of primary gastric adenocarcinoma, the concentrations of TGF-β1 in the cancer tissue were significantly higher compared with surrounding normal tissues, though in the cancer tissue the concentration of active TGF-β1 varied from 1.6 to 81.3 pg/mg of protein whereas total TGF-β1 levels ranged from 21.1 to 620.1 pg/mg of protein [15]. Higher concentrations of TGF-β1 in the gastric adenocarcinoma tissue were linked with shorter patient survival, perhaps due to the patient-derived factors, i.e. higher body mass index (BMI) [16] which is associated with increased levels of adipose tissue-derived inflammatory factors and increased levels of adipose tissue-derived TGF-β1 and PAI1 [17]. It is worth highlighting that higher concentrations of an active form of a cancer-derived form of TGF-β1 in patients with gastric adenocarcinoma correlated positively with urokinase (uPA) activity [15], supporting the predominant role of plasmin in TGF-β activation in the tumour microenvironment postulated in this review. Indeed, higher rates of plasmin-dependent activation of cancer-derived TGF-β would then dose-dependently contribute to increased secretion of PAI1 [18] to constrain excessive TGF-β signalling in the tumour microenvironment, although at the cost of increased risk of thrombotic events. In this context, increased PAI-1 secretion might even have beneficial effects in cancer as it would inhibit cancer progression by comprising TGF-β signalling. Indeed, it was shown that weak PAI1 expression was an independent positive marker for lymph node metastases (8-fold higher risk) when compared to strong PAI-1 expression associated with better prognosis of oral cancer patients [19].

Therefore, the most negative scenario for cancer patients would be driven by high concentrations of active cancer-derived TGF-β. This seems to be a feature of pancreatic cancer which, among other types of cancer, is associated with the highest rates of CAT events [5]. In the progression of pancreatic cancer, activated stellate cells (the resident cells of the pancreas), secrete high levels of TGF-β1 that not only contribute to the development of pancreatic adenocarcinoma but also lead to its more aggressive phenotype [20]. These very high amounts of cancer-derived TGF-β in pancreatic cancer patients could account for high rates of CAT incidence [5] as they simultaneously yield high levels of PAI1 and PAI1 activity [21]. Such a negative scenario (i.e. high concentrations of cancer-derived form of TGF-β) could also be true for late-stage disseminated cancers with distant metastases. Since metastatic cancer cells secrete high amounts of PAI1 [22], it could not only indicate high rates of TGF-β signalling [18] but could also directly account for increased rates of CAT events reported for metastatic cancers [7].

As implied above, the TGF-β cytokine is secreted in the latent form and must be activated to exert its biological effects [23]. In the pro-coagulant cancer microenvironment, overactivation of platelets is associated with increased incidence of thrombus formation, which is followed by plasmin generation and activation of fibrinolysis [24]. Therefore, the pro-coagulant cancer microenvironment is associated with increased fibrinolysis rates that simultaneously facilitate plasmin-dependent activation of latent TGF-β and, in conditions of high fibrinolysis rates, plasmin-dependent activation of TGF-β might become the predominant activation mechanism of TGF-β cytokine, as was shown in a case of gastric cancer [15]. Consequently, excessive activation of TGF-β (derived from cancer cells and the tumour microenvironment) by plasmin would lead to increased PAI1 secretion and inhibition of fibrinolysis, thus permanently increasing the risk of thrombosis in cancer patients (Fig. 1B). The direct link between TGF-β1 and the higher risk of thrombosis has already been confirmed. Specifically, TGF-β1 deficiency in platelets significantly compromised venous thrombus formation (fibrin-rich), although it did not alter arterial thrombus formation (fibrin-poor and consisting mainly of platelets) [25]. Moreover, TGF-β1 released from platelets contributed to hypercoagulability in veno-occlusive disease after hematopoietic stem cell transplantation [26]. Finally, the absence of TGF-β1 in platelets was associated with faster venous thrombus resolution, whereas deleting TGF-βRII in endothelial cells resulted in larger and more fibrotic venous thrombi because it increased circulating active TGF-β1 levels, endothelial TGFβRI/ALK1 (activin receptor-like kinase), and TGFβRI/ALK5 expression [27].

However, it should be mentioned that excessive TGF-β signalling would also accelerate progression of cancer by other mechanisms, such as promotion of tumour stemness and cancer drug resistance via the epithelial-mesenchymal transition (EMT) of cancer cells [28], and promotion of endothelial cell migration, proliferation, and angiogenesis via induction of EndMT [29]. The prominent role of TGF-β in promoting cancer progression via EMT/EndMT induction or immunosuppression is beyond the scope of this review and has been reviewed in detail elsewhere [30].

##  The serine protease inhibitor (serpin) superfamily member PAI-1

The major source of PAI-1 in the blood are platelets [31]. Therefore, antiplatelet therapy effectively reduces plasma PAI1 levels [32, 33] (Table 1), similar to anticoagulant treatment [34–37] (Table 1), most likely by limiting platelet activation. In conditions of anticoagulant/antiplatelet treatment, lower PAI1 levels would favour plasmin-dependent activation of the excess of cancer-derived TGF-β secreted by cancer cells and the cells in cancer microenvironment (Fig. 1C).

**Table 1:** Anticoagulant/antiplatelet regimens used to treat CAT. Irrespective of the mechanism of anticoagulant/antiplatelet action, the pharmacological agents used to treat or prevent CAT lowered the level and/or activity of PAI1

Treatment	Mechanism of action	Effects of therapy on PAI1
Parenteral anticoagulants LMWHs	Inhibit factor Xa.	Enoxaparin, dalteparin and nadroparin significantly lowered PAI1 levels [34].
Parenteral anticoagulants unfractionated heparin (UFH)	Binds to antithrombin and platelets, inhibits factor Xa.	UFH decreased PAI1 levels [35].
Parenteral anticoagulants fondaparinux	Inhibits factor Xa.	Fondaparinux lowered PAI1 concentration in the plasma [41].
Oral anticoagulants vitamin K antagonists (VKAs, e.g. warfarin)	Prevent gamma-carboxylation of vitamin K-dependent coagulation factors.	Warfarin prevented the postoperative rise in activity of plasminogen activator inhibitor (PAI) [42].
Oral anticoagulant: non-vitamin K oral anticoagulants DOACs Factor Xa inhibitors (rivaroxaban, apixaban, edoxaban) Factor IIa and/or direct thrombin inhibitors (dabigatran)	Inhibit either factor Xa or thrombin.	Edoxaban decreased PAI1 plasma levels [36]. Rivaroxaban decreased PAI1 expression [37].
Next-generation anticoagulants: Factor XI (FXI) inhibitors (abelacimab)	Abelacimab binds to catalytic domain of FXI and locks it in the zymogen confirmation, preventing its activation by FXIIa and thrombin.	The efficacy and safety of abelacimab in the treatment of CAT (VTE) is currently under investigation (ASTER) [43].
Oral antiplatelet therapy: Aspirin	Aspirin inhibits prostaglandin production in platelets.	Aspirin reduced platelet-derived PAI1 levels [32].
Oral dual antiplatelet therapy (DAPT): Aspirin and P2Y12 inhibitors	Combines aspirin-mediated inhibition of prostaglandin production in platelets with platelet P2Y12 receptor blockade.	Ticagrelor or prasugrel with aspirin reduced plasma PAI1 levels even more potently than clopidogrel with aspirin or aspirin alone [33].

While the majority of platelet-derived PAI1 is in the inactive form [31], platelets can also *de novo* synthesise PAI1, most of which is in the active conformation as it complexes with tPA, inhibiting tPA activity and, possibly, that of uPA [38]. The *de novo* synthesis of an active form of platelet-derived PAI1 can be potentiated by thrombin [38]. It remains to be confirmed whether the release of the active PAI1 from circulating platelets can also be induced by TGF-β in the self-regulatory mechanism shown in Fig. 1A. This is plausible since platelets express functional TGF-β receptors and the intracellular effectors of TGF-β signalling [39]. One way or another, the secretion of the platelet-derived active form of PAI1 could be an important mechanism constraining the overactivation of deleterious TGF-β signalling in the pro-coagulant cancer microenvironment, which, if inhibited by antiplatelet/anticoagulant therapy, could drive CAT and cancer progression.

The other major source of PAI1 in the blood are endothelial cells, though endothelium-derived circulating PAI1 levels are substantially lower than platelet-derived PAI1 levels [31]. Consequently, as mentioned above, deleting TGF-βRII in endothelial cells resulted in larger, more fibrotic, and more highly vascularised venous thrombi concomitantly with increased endothelial TGF-βRI/ALK1 (activin receptor-like kinase) and TGF-βRI/ALK5 expression and higher circulating active TGF-β1 levels [27]. PAI1 can also originate from other cell types such as vascular smooth muscle cells [40]. Intriguingly, some types of cancer cells can also increase the expression of PAI1 [19, 22].

## Could anticoagulant/antiplatelet treatment facilitate the activation of cancer-derived TGF-β?

Many anticoagulant/antiplatelet approaches are currently used to treat and/or prevent CAT (Table 1). This group comprises low molecular weight heparins (LMWHs), direct oral anticoagulants (DOACs), aspirin, and other agents. The need for long-term anticoagulant/antiplatelet treatment in cancer patients at high risk of, or with ongoing, CAT events is indisputable. Intriguingly, there is literature evidence that some anticoagulant/antiplatelet regimens could accelerate cancer progression in animal models [44–47] and increase the number of cancer deaths in humans [48–53] if used for extended periods.

Accordingly, long-term dual antiplatelet therapy with clopidogrel and aspirin increased mortality of mice with metastatic breast cancer [45], and adverse effects on breast cancer progression were also observed for mice treated with aspirin alone [44]. Additionally, dabigatran [46], ximelagatran, and hirudin [47] increased the number of lung metastases, which was associated with increased lung endothelium permeability.

Considering the human population, the DAPT (Dual Antiplatelet Therapy) trial found that long-term antiplatelet therapy with clopidogrel and prasugrel supplemental to aspirin was associated with increased cancer diagnoses and, thus, increased non-cardiovascular-related deaths in patients [48, 53]. These results were consistent with the TRITON trial comparing the effects of prasugrel and clopidogrel in patients with acute coronary syndromes [48, 51], and the TRACER trial investigating the effects of thrombin receptor antagonist vorapaxar on clinical event reduction in acute coronary syndrome [48, 54]. The potential link between antiplatelet agents, anticoagulants, and excess cancer-related deaths was also reported for apixaban (APPRAISE-2 trial) and ticagrelor (PEGASUS trial), and was considered controversial in the PLATO trial [49]. Finally, higher mortality attributed to cancer-related death was observed among ostensibly healthy older adults who received daily aspirin on a long-term basis (ASPREE trial) [50]. The mechanistic rationale for these unexpected but alarming findings remains unknown but some of the patients with cardiovascular diseases participating in these trials may have already harboured occult cancers, the progression of which could have been accelerated with anticoagulant/antiplatelet therapy. This scenario seems possible as the time of treatment was rather too short for new cancers to develop as a result of the anticoagulant/antiplatelet therapy alone [52].

What might be the mechanism of the accelerated progression of these occult cancers? In our previous work, we proposed that the increased number of cancer cases detected in the clinical trials described above may be associated with inhibition of platelet-dependent protection of endothelial barrier integrity by anticoagulant/antiplatelet treatment [55]. One of the mechanisms of platelet-dependent protection of endothelial barrier integrity might, paradoxically, be platelet-derived PAI1 that, in conditions of a hypercoagulable cancer microenvironment, could counteract the excessive activation of cancer-derived TGF-β that can disrupt endothelial barrier integrity [56]. This provocative hypothesis is not unsubstantiated since inhibition of PAI1 increases endothelial barrier permeability both *in vitro* and *in vivo* [57] and TGF-β signalling was approximately threefold higher in cells with PAI1 knocked out [58]. Accordingly, in cancer conditions, anticoagulant/antiplatelet treatment that inhibits platelets, (thus reducing the availability of PAI-1 in the circulation) would facilitate plasmin-dependent activation of cancer-derived TGF-β (Fig. 1C) which, in turn, would potentiate deleterious TGF-β signalling and drive cancer progression.

## Future perspectives

If the hypothesis regarding increased plasmin-dependent activation of cancer-derived TGF-β (comprising TGF-β derived from cancer cells and the tumour microenvironment) by anticoagulant/antiplatelet treatment were true, it may be the major mechanism of thrombosis in cancer patients undermining the effectiveness and safety of anticoagulant/antiplatelet treatment. In such conditions, combining anticoagulant/antiplatelet treatment and anti-TGF-β therapy might provide substantial benefits. Such an approach might not only effectively lower thrombotic risk but also, in a longer-term perspective, prevent accelerated cancer progression, both of which are driven by TGF-β signalling. Indeed, to date, many therapeutic approaches to attenuate deleterious TGF-β effects in cancer have been tested and found to be beneficial for cancer patients [59], including inhibition of hyperactive TGF-β signalling to overcome patient resistance to immunotherapy [60]. Some of these already-known anti-TGF-β therapies could be now easily tested in the treatment of cancer patients undergoing anticoagulant/antiplatelet treatment.

## Conclusions

Despite the introduction of anticoagulant/antiplatelet therapy into the treatment of cancer patients experiencing CAT, the thrombotic risk, the probability of recurrent CAT events, and the mortality rates of patients with cancer still remain very high [13]. This phenomenon might be associated with increased rates of plasmin activity evoked by anticoagulant/antiplatelet treatment that would allow for excessive activation of cancer-derived TGF-β overproduced by cancer cells and the tumour microenvironment. In turn, increased TGF-β signalling would drive PAI1 expression and secretion resulting in the inhibition of fibrinolysis and increased thrombotic risk. Therefore, it is an urgent medical need to validate the hypothesis that anticoagulant/antiplatelet therapy for cancer patients on a long-term basis is linked to potentiated TGF-β signalling. If this hypothesis is true, it would form a basis for investigating the effectiveness of combined anticoagulant/antiplatelet regimens and anti-TGF-β therapy to make anticoagulant/antiplatelet therapy of cancer patients experiencing CAT more effective and safe.

## Data Availability

No datasets were generated or analysed during the current study.
